# Interpretable machine learning for urothelial cells classification and risk scoring in urine cytology

**DOI:** 10.1016/j.isci.2025.114259

**Published:** 2025-11-27

**Authors:** Lei Xiong, Xinyi Cao, Yu Shang, Zongyue Lu, Hao Jiang, Chang Shi, Chengzhi Zhang, Zhongjing Ma, Lili Tian, Xiaojie Wang, Jiwei Liu, Jia Li, Fengqi Fang

**Affiliations:** 1 Department of Oncology, The First Affiliated Hospital of Dalian Medical University, Dalian, China; 2 Department of Medical Development, Hangzhou Zhiwei Information and Technology Co., Ltd., Hangzhou, China; 3 Department of Pathology, The First Affiliated Hospital of Dalian Medical University, Dalian, China

**Keywords:** health sciences, cancer, machine learning

## Abstract

Urine cytology is widely used for detecting urothelial carcinoma (UC), though its performance is constrained by limited sensitivity and substantial interobserver variability. An interpretable machine learning framework was developed to classify urothelial cells and to estimate slide-level risk of high-grade UC. 10,230 expert-annotated urothelial cells were used to extract 20 quantitative feature representing cytomorphologic criteria defined by the Paris System. Ordinal logistic regression and random forest models were trained and validated, achieving over 90% accuracy for classifying cells into normal, atypical, or suspicious categories. Interpretable morphological features were identified as major contributors to prediction. Slide-level risk scores were derived from aggregated cell probabilities in a validation set of 247 cases. These scores effectively stratified negative, atypical, low-grade, and high-grade UC cases (*p* < 0.0001). Through alignment with established cytologic criteria, this feature-based framework provides a transparent and quantitative approach that may improve consistency, efficiency, and interpretability in digital urinary cytology.

## Highlights

• An interpretable machine learning framework analyzes urothelial cells in urine cytology
• Quantitative cytologic features reflecting diagnostic criteria are systematically extracted
• Transparent models achieve high cell classification accuracy with explainable features
• Slide level risk scores stratify cytology cases by likelihood of high-grade carcinoma

## Graphical abstract

**Figure:**
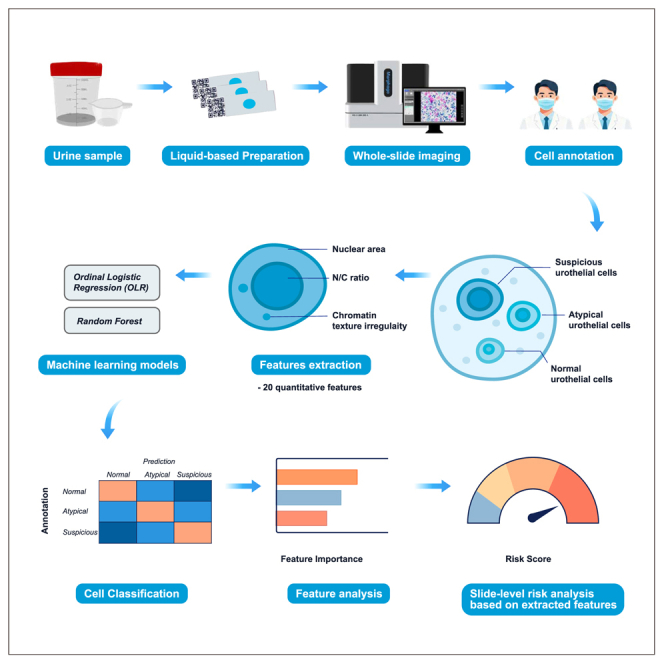

## Introduction

Urine cytology is a valuable noninvasive method for detecting urothelial carcinoma (UC), especially high-grade tumors. However, it faces persistent limitations in sensitivity and interpretive consistency. Traditionally, cytopathologists assess cellular morphology to identify malignancy, but considerable interobserver variability exists, particularly in equivocal cases such as atypical urothelial cells (AUCs), which exhibit some worrisome yet insufficient diagnostic features.^1^

To address these challenges, the Paris System for Reporting Urinary Cytology (TPS), introduced in 2016,^2^ standardized diagnostic terminology and criteria, with an emphasis on features associated with high-grade urothelial carcinoma (HGUC). TPS provides clear morphologic definitions—such as large, hyperchromatic nuclei, increased nuclear-to-cytoplasmic (N/C) ratio, and irregular chromatin—for classification into categories including negative for HGUC (NHGUC), AUC, suspicious for HGUC (SHGUC), and HGUC. Each category carries an estimated risk of malignancy and corresponding clinical recommendations.^3^ The adoption of TPS has improved reporting consistency and predictive value for HGUC.^4^ Nonetheless, urine cytology remains constrained by suboptimal sensitivity, with many studies reporting detection rates of only 50%–75% for high-grade tumors, though specificity remains near 98%.^4^^,^^5^ This performance gap and continued reliance on subjective visual interpretation have motivated research into adjunctive artificial intelligence (AI) tools for urine cytology.

Recent advances in digital pathology and AI have created opportunities for enhancing cytological analysis.^6^ Prior studies have demonstrated the potential of data-driven algorithms to automate detection of UC.^7^^,^^8^^,^^9^^,^^10^^,^^11^ The algorithms can analyze digitized cytology slides to identify abnormal cells or patterns that correlate with malignancy.^12^ For example, Kaneko et al. developed a convolutional neural network (CNN) that classified single-cell images as “benign” vs. “atypical/malignant” with accuracy exceeding 90%, surpassing average human performance.^13^ Similarly, Wu et al. introduced a multistage AI pipeline combining cell detection, feature extraction, and CNN classification, which significantly improved the sensitivity of cytology for detecting UC in a multicenter study.^14^ Tsuji et al. developed an automated AI system to assist in slide-level prediction of HGUC and reached a sensitivity of 63%.^10^ However, deep learning models operate as “black boxes”—their decision-making processes are based on abstract, high-dimensional features not directly interpretable by humans.^15^ This lack of transparency poses a challenge for clinical adoption and regulatory approval, where explainability is increasingly emphasized. Explainable AI (XAI) solutions aim to provide interpretable reasoning for their predictions.^16^ For cytology, this includes highlighting which features—such as increased nuclear size or coarse chromatin—led to a cell being flagged as AUC or suspicious.^17^^,^^18^

In this work, we developed a feature-based, interpretable classification system for urothelial cells in urine cytology. Instead of a black-box neural network, we extracted quantitative morphological and textural features from individual cells and used machine learning (ML) models. The features were chosen to reflect known cytologic criteria from TPS (such as N/C ratio, nuclear size, chromatin texture, and nuclear shape). Our aims were: (1) to achieve accurate cell-level classification into normal, degenerated, atypical, or high-grade carcinoma categories with a transparent model, (2) to interpret the model’s decisions in terms of familiar cytological features, and (3) to explore aggregation of cell-level results into a whole-slide risk score that could predict case-level likelihood of HGUC. By providing cell-level explanations and a slide-level risk index, this approach could assist cytopathologists in screening urine cytology slides, flagging high-risk cases and explaining the rationale with objective feature values. We envision this interpretable model as an augmentative tool in digital cytopathology workflows, improving consistency and throughput in environments where expert review is limited.

## Results

### Feature analysis

**Figure 1:**
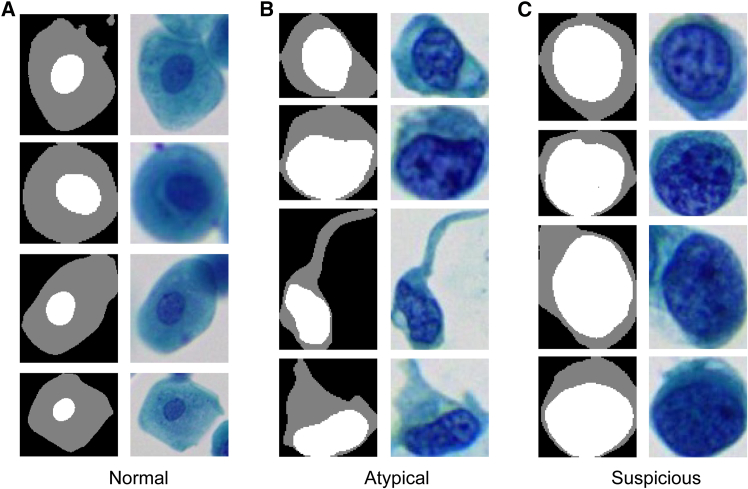
Representative urothelial cells and their segmentations (masks with nucleus in white and cytoplasm in gray) (A) Normal superficial urothelial cells with small round nuclei. (B) Atypical cells with enlarged, mildly irregular nuclei. (C) Suspicious urothelial cells for HGUC with irregular hyperchromatic nuclei.

**Figure 2:**
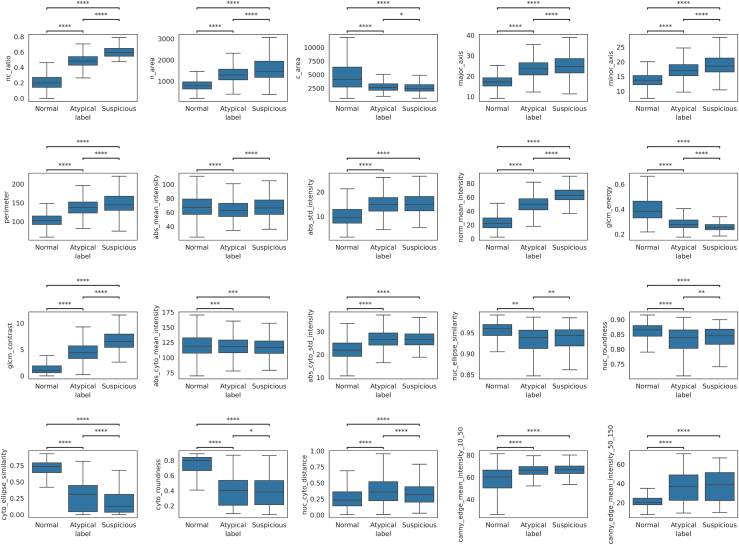
Distribution of 20 quantitative features across normal, atypical, and suspicious urothelial cells categorized for HGUC assessment Morphological features include nuclear area (*n_area*), cell area (*c_area*), N/C ratio (*nc_ratio*), nuclear major and minor axis lengths (*major_axis* and *minor_axis*), and nuclear perimeter (*perimeter*). Nuclear and cytoplasmic shape descriptors include ellipse similarity (*nuc_ellipse_similarity* and *cyto_ellipse_similarity*) and roundness (*nuc_roundness* and *cyto_roundness*). Nuclear-cytoplasmic displacement is quantified by *nuc_cyto_distance*. Intensity features include absolute and normalized nuclear mean intensity (*abs_mean_intensity* and *norm_mean_intensity*), nuclear intensity standard deviation (*abs_std_intensity*), cytoplasmic mean and standard deviation (*abs_cyto_mean_intensity* and *abs_cyto_std_intensity*). Texture features include GLCM energy and contrast (*glcm_energy* and *glcm_contrast*). Edge features quantify nuclear edge sharpness using Canny filter mean responses at two thresholds: 10–50 (*canny_edge_mean_intensity_10_50*) and 50–150 (*canny_edge_mean_intensity_50_150*). Boxplots are stratified by cytological class (normal, atypical, and suspicious). Data are represented as median with interquatile range. Statistical differences were evaluated using pairwise *t* tests with Bonferroni correction; asterisks indicate significance levels (∗∗∗∗*p* < 0.0001, ∗∗∗*p* < 0.001, ∗∗*p* < 0.01, ∗*p* < 0.05).

**Figure 3:**
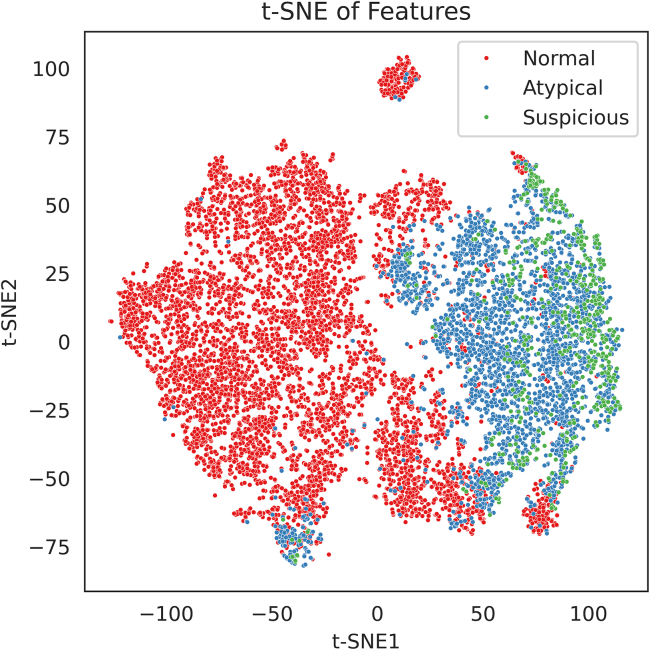
Two-dimensional t-SNE visualization of single-cell feature space Each point represents one cell in the dataset, embedded into two dimensions using t-SNE based on the 20 extracted features. The t-SNE plot illustrates partial separation between the cell types. Normal cells formed a tight cluster in one region of the map, indicating relatively homogeneous feature profiles for benign urothelial cells. Suspicious cells clustered predominantly in a separate area, distant from the normal cluster, consistent with their distinct morphologic feature patterns. Atypical cells were dispersed between the normal and suspicious clusters, overlapping with both. This continuity is in line with the cytological progression of urothelial atypia, where atypical cells share some features with benign cells and some with malignant cells.

Based on segmented urothelial cells (Figure 1), 20 features were extracted. Boxplots show the values of each quantitative feature for normal, atypical, and suspicious urothelial cells (Figure 2). Each box’s central line denotes the median, with the box spanning the interquartile range (IQR) and whiskers extending to 1.5×IQR. Significant differences between cell categories are indicated (∗∗∗∗*p* < 0.0001, ∗∗∗*p* < 0.001, ∗∗*p* < 0.01, ∗*p* < 0.05, two-sample *t* tests with Bonferroni correction). Overall, many cytomorphometric features exhibited progressive changes from normal to suspicious cells. For example, suspicious cells showed markedly higher N/C ratios and larger nuclear size compared to normal cells (all *p* < 0.0001). “Gray level co-occurrence matrices (GLCM) contrast” was significantly elevated in atypical and suspicious cells (*p* < 0.0001), reflecting stronger gray-level transitions and more complex chromatin texture. In contrast, total cell area did not significantly differ between atypical and suspicious cells (*p* < 0.05), suggesting that suspicious cells often have proportionally larger nuclei without much increase in overall cell size. “Abs_mean_intensity” showed a statistically significant difference across cell categories (∗∗∗∗*p* < 0.0001), but the distributions largely overlapped. Median values and IQRs were similar among normal, atypical, and suspicious cells, suggesting that although hyperchromasia is a known cytological marker, the absolute grayscale intensity alone may not provide strong discriminative power in practice. Additionally, to explore the overall distribution of the 20 morpholigical and textural feature, t-distributed Stochastic Neighbor Embedding (t-SNE) analysis was applied (Figure 3).

### Model performance

**Figure 4:**
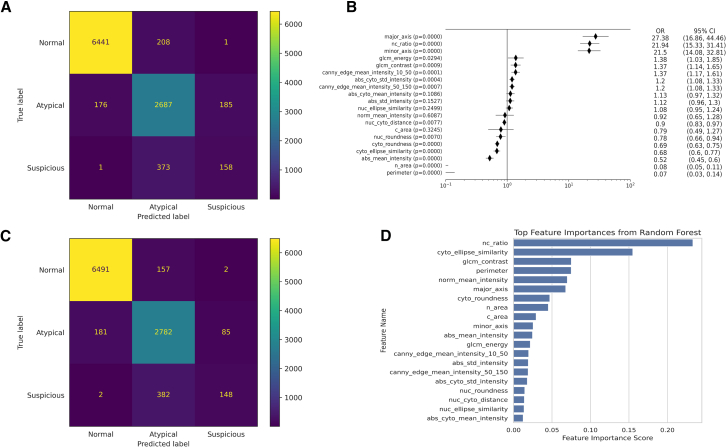
Feature interpretability and prediction outputs of OLR and RF models for single-cell cytological classification (A) Confusion matrix of the OLR model. (B) Odds ratios of 20 features from the OLR model. Data are shown as mean ± standard deviation across five cross-validation folds. (C) Confusion matrix of the RF model. (D) Feature importance ranking from the RF model.

Figure 4 displays the results from both models. Two ML models, ordinal logistic regression (OLR) and random forest (RF), were trained on the single-cell feature dataset to classify urothelial cells into normal, atypical, or suspicious categories. The confusion matrices reflect predictions from the entire annotated dataset of 10,230 cells, aggregated across all 5-folds of cross-validation. This evaluation strategy allowed each cell to be independently tested and included in the final metrics.

The OLR model achieved an overall accuracy of 0.9077 (Table 1). Normal cells were classified with high sensitivity (0.9686) and positive predictive value (PPV) (0.9733), resulting in an F1-score of 0.9709. For atypical cells, the sensitivity and PPV were 0.8816 and 0.8222, respectively. Classification performance for suspicious cells was lower, with sensitivity of 0.2970 and PPV of 0.4593. Feature effect estimates from the OLR model (Figure 4B) showed that the most influential predictors were nuclear major axis length (OR = 27.38), N/C ratio (odds ratio [OR] = 21.94), and nuclear minor axis length (OR = 21.50), all with *p* < 0.0001. These features reflect the importance of nuclear enlargement in risk stratification. Additional texture features such as GLCM contrast and nuclear edge sharpness were also positively associated with higher-risk categories.

**Table 1:** Performance metrics of OLR and RF models for urothelial cell classification

Model	OLR					RF				
Class	Sensitivity	Specificity	PPV	NPV	F1-score	Sensitivity	Specificity	PPV	NPV	F1-score
Normal	0.9686	0.9506	0.9733	0.9421	0.9709	0.9761	0.9489	0.9726	0.9553	0.9743
Atypical	0.8816	0.9191	0.8222	0.9481	0.8509	0.9127	0.925	0.8377	0.9615	0.8736
Suspicious	0.2970	0.9808	0.4593	0.9622	0.3607	0.2782	0.991	0.6298	0.9616	0.3859
Accuracy	0.9077					0.9209				

The RF model achieved a slightly higher overall accuracy of 0.9209. Sensitivity and PPV for normal cells were 0.9761 and 0.9726, respectively. For atypical cells, sensitivity was 0.9127 and PPV was 0.8377. Suspicious cells had a sensitivity of 0.2782 and PPV of 0.6298. Feature importance rankings (Figure 4D) corroborated the dominance of N/C ratio and chromatin heterogeneity in classification. Both models consistently highlighted interpretable cytomorphologic parameters as key discriminative features in urothelial cell classification.

### Slide-level risk scoring

**Figure 5:**
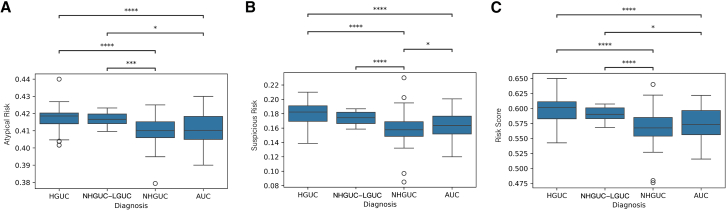
Slide-level risk score distributions by cytological diagnostic category Boxplots depict the distribution of model-derived risk scores for each diagnostic group: NHGUC, AUC, NHGUC-LGUC, and HGUC. (A) Suspicious risk: average probability of the suspicious urothelial cells per slide. (B) Atypical risk: average probability of the AUCs per slide. (C) Risk score: average probability of atypical and suspicious urothelial cells per slide. Data are represented as boxplots showing median with IQR. Statistical significant was determined using *t* tests with Bonferroni correction (∗∗∗∗*p* < 0.0001, ∗∗∗*p* < 0.001, ∗∗*p* < 0.01, ∗*p* < 0.05).

Slide-level risk scores were computed by averaging RF model-predicted probabilities of atypical and suspicious urothelial cells across each whole slide image (WSI). The distributions of these scores across diagnostic categories—NHGUC, AUC, NHGUC-LGUC, and HGUC—are shown in Figure 5. Atypical risk scores (Figure 5A) showed a graded increase from NHGUC to AUC, NHGUC-LGUC, and HGUC, reflecting rising atypicality across categories. Suspicious risk scores (Figure 5B) were significantly elevated in slides diagnosed as HGUC compared to all other groups (∗∗∗∗*p* < 0.0001), indicating a higher burden of cells with high predicted probability of malignancy. The combined risk score (Figure 5C), aggregating both atypical and suspicious probabilities, demonstrated the clearest stratification, with HGUC slides exhibiting significantly higher scores than NHGUC and AUC (∗∗∗∗*p* < 0.0001).

## Discussion

In this study, we present an interpretable machine learning framework that addresses long-standing challenges in urine cytology, notably the subjectivity of cell interpretation and the limited sensitivity for detecting HGUC. Traditional urine cytology is highly specific (∼98%) but often only detects about 50%–75% of HGUC cases, due to subtle morphologic changes and significant interobserver variability in borderline cases. By contrast, our feature-based approach applies objective quantitative criteria to each cell, yielding reproducible classifications that reduce reliance on subjective judgment. The models achieved high cell-level accuracy (∼90%–92%), with especially strong performance in identifying normal and atypical cells (e.g., the OLR model’s sensitivity was ∼0.97 for normal cells and ∼0.88 for atypical cells). This is notable given that distinguishing true atypia from benign reactive changes is a common source of diagnostic inconsistency in manual cytology. Although classification of suspicious (high-grade) cells remained challenging (the RF model’s sensitivity for this class was ∼0.28), the overall accuracy and consistency of our pipeline suggest it can augment baseline cytology performance and potentially improve detection rates for UC in practice.

A key advantage of our framework is its interpretability, effectively addressing the “black-box” issue often associated with deep learning models in cytology. Instead of relying on abstract, high-dimensional features, our OLR and RF models use well-defined morphological and textural attributes that are familiar to cytopathologists. The most influential predictors identified by the OLR, such as nuclear major axis length, N/C ratio, and nuclear minor axis length, had large effect sizes (odds ratios > 20) and highlight the hallmark finding of nuclear enlargement in urothelial atypia. These parameters have well-established diagnostic significance: increased nuclear size and N/C ratio are recognized as critical indicators of high-grade lesions,^18^^,^^19^^,^^20^ coarse and heterogeneous chromatin texture reflects chromatin clumping and genomic instability associated with malignant transformation,^21^^,^^22^ and irregular nuclear membranes are reliable morphological discriminators of carcinoma.^22^^,^^23^ Similarly, the inclusion of chromatin texture metrics (e.g., Gray-Level Co-occurrence Matrix contrast and nuclear edge sharpness) among the top features corresponds to the coarse or irregular chromatin often observed in malignant cells.^24^^,^^25^ By capturing these well-understood cellular features in quantitative form, our interpretable model aligns computational prediction with cytopathologists’ diagnostic reasoning, thereby promoting transparency, reproducibility, and clinical trust.

In terms of classification performance, our approach compares favorably with reported values in both traditional cytology and prior AI-based studies. The overall accuracy of ∼91% (OLR) to ∼92% (RF) in differentiating normal, atypical, and suspicious cells is on par with the best results from deep learning classifiers. For example, Kaneko et al. reported >90% accuracy for a CNN distinguishing benign vs. malignant cells.^13^ However, unlike a black-box CNN, our models maintain interpretability while handling a more granular three-class problem. Notably, the sensitivity for atypical cells in the RF model reached ∼0.91, which is encouraging given that urine cytology’s sensitivity for high-grade cancer is typically only ∼50–75% at the case level. This suggests our method could detect a higher proportion of clinically significant atypia than cytologists might on average, without sacrificing specificity (indeed, our models showed excellent precision for Normal cells, reflecting low false-positive rates). Wu et al. similarly aimed to bolster cytology sensitivity using a multistage AI pipeline, underscoring that improving detection of HGUC is a priority in the field. Our results reinforce that machine learning can enhance sensitivity; importantly, we achieve this with a transparent, feature-based model that could be more readily integrated into clinical practice.

Beyond individual cell classification, our study uniquely incorporates a slide-level risk scoring system. This approach proved effective in stratifying cases: slides diagnosed as HGUC had significantly higher model-derived risk scores compared to negative or atypical cases (with HGUC slides showing the highest risk indices, ∗∗∗∗*p* < 0.0001). In other words, even when malignant cells were relatively scarce on a slide, their contributions to the overall risk metric were sufficient to distinguish high-grade cases. Such a slide-level index has practical implications for workflow optimization. It could be used as an automated triage tool to flag high-risk specimens for rapid review or ancillary testing, ensuring that slides likely harboring HGUC receive prompt attention. Conversely, cases with very low-risk scores might be candidates for expedited sign-out as negative, thus improving laboratory efficiency. We anticipate that this risk-based prioritization, combined with the model’s ability to explain *why* a slide is high-risk (e.g., due to a preponderance of cells with large nuclei or irregular chromatin), can augment cytology screening protocols. As others have suggested, integrating AI into cytopathology can improve consistency and throughput; our slide-level risk metric is a concrete step in that direction, providing a safety net for missed cancers while also elucidating the cytologic features underlying the risk.

Recent advances in ancillary molecular testing, particularly DNA-methylation-based urine assays, have demonstrated remarkable sensitivity for detecting bladder cancer from urine samples and offer complementary diagnostic insights that can augment cytology. Many studies^26^^,^^27^^,^^28^^,^^29^ have highlighted the potential of urinary DNA methylation test for identifying urothelial malignancy. These molecular tests offer high sensitivity but are often cost-intensive and require specialized laboratory infrastructure. In contrast, our interpretable ML-based cytology framework provides an immediate, image-driven assessment that can be seamlessly integrated into existing digital cytopathology workflows without additional reagents or molecular testing. As such, the proposed slide-level risk score could serve as a triage tool to prioritize specimens for ancillary molecular testing combining the efficiency of image-based screening with the molecular precision of advanced assays.

This study presents an interpretable machine learning framework for classifying urothelial cells in liquid-based urine cytology. By extracting quantitative morphologic and textural features aligned with established cytological criteria, our models achieved high accuracy in distinguishing normal, atypical, and suspicious cells, while maintaining clinical transparency. Moreover, the proposed slide-level risk score effectively stratified cases by HGUC risk, offering a promising tool for cytology triage. Future work will involve prospective validation using histopathological gold standards and integration into real-world digital pathology workflows.

### Limitations of the study

Despite these promising results, there are important limitations in our study. First, the present study used expert cytological consensus as the reference standard for model training and validation, rather than histopathologically confirmed diagnoses. Although cytologist consensus provides a practical and widely accepted surrogate ground truth in retrospective cytology studies, it introduces a degree of circularity. This may partly explain the model’s low sensitivity for the “suspicious for HGUC” cell class, since the true biological status of these cells was uncertain and the category itself represents an area of diagnostic ambiguity in routine cytology. Future work will therefore incorporate paired histopathological follow-up to provide a definitive gold standard for model refinement and validation. Second, although over 10,000 individual cells were analyzed, these originated from only 50 patients, which limit the morphological diversity of the training cohort. Urothelial cytology exhibits broad heterogeneity related to patient demographics, specimen collection methods, and tumor biology. Expanding the dataset to include a larger and more diverse patient population is essential for improving generalizability and reducing sampling bias. Multi-institutional data aggregation will also help the model adapt to variations in staining, imaging, and slide preparation that occur across laboratories. Third, degenerated or poorly preserved cells were excluded from training to ensure clear morphologic feature extraction. However, in routine practice, such imperfect cells are frequently encountered and often represent diagnostic challenges. A clinically robust AI tool must be capable of handling these real-world variations. Finally, while the current results demonstrate high overall accuracy, the model’s clinical value depends on reliably identifying rare high-risk cells that may indicate malignancy. Addressing this class imbalance through targeted data augmentation, improved sampling of rare events, and refined feature engineering will be central to the next phase of research. By explicitly recognizing these limitations, we aim to position this work as an early but necessary step toward developing an interpretable, clinically deployable AI system for urinary cytology. Future validation using histologically confirmed outcomes and broader, heterogeneous datasets will be key to demonstrating real-world diagnostic impact and clinical adoption.

## Resource availability

### Lead contact

Further information and requests for resources should be directed to and will be fulfilled by the lead contact, Fengqi Fang (18098876723@163.com).

### Materials availability

We have established urothelial image datasets (normal, atypical, and suspicious) in this study. The datasets are used to train and evaluate the model.

### Data and code availability

• Data will be made available from the lead contact on request.
• All original code has been deposited at Github and is publicly accessible from the publication date. The DOI is listed in the key resources table.
• Any additional information required to reanalyze the data reported in this paper is available from the lead contact upon request.

## Acknowledgments

This work was supported by the Science and Technology Program of Liaoning Province under grant number 2023JH2/101300110.

## Author contributions

L.X., X.C., and Y.S. contributed equally to this work and should be considered co-first authors. L.X. designed the study, supervised the overall project, and contributed to manuscript writing. X.C. led the machine learning pipeline development, performed data analysis and model interpretation, and contributed to manuscript writing. Z.L. contributed to model development, feature extraction, and data visualization. H.J., C.Z., Z.M., L.T., X.W., and J.L. contributed to dataset preparation and clinical data curation. Y.S. and C.S. performed expert cytological review and annotation of urothelial cells, providing diagnostic interpretation of cytology slides according to the Paris System criteria. Their domain expertise was essential for establishing high-quality ground truth for model training and evaluation. J.L. and F.F. conceived the study, provided guidance on clinical significance, and supervised the entire project. They also contributed to manuscript revision and are the corresponding authors of this work.

## Declaration of interests

X.C. and Z.L. are employees of Hangzhou Zhiwei Information and Technology Co., Ltd., which provided technical support and scanning equipment for this study.

## STAR★Methods

### Key resources table

**Table:** 

REAGENT or RESOURCE	SOURCE	IDENTIFIER
**Deposited data**		

Source Code	This paper	https://github.com/zongyue-lu/interpretable_urocell_ml.git
Dataset	This paper	N/A

**Software and algorithms**		

Morphogo-U System	This paper	https://www.morphogo.com/en/
Python 3.9	Python Software	https://www.python.org/
Microsoft Excel 2020	Microsoft	https://www.microsoft.com/
GraphPad Prism 10	GraphPad Software	https://www.graphpad.com

### Experimental model and study participant details

#### Human samples and ethical approval

This study was conducted on a dataset of 297 urine cytology cases obtained from pathology archives with appropriate ethics approval in the First Affiliated Hospital of Dalian Medical University (Ethics Approval No. PJ-KS-KY-2024-641). Given the retrospective design and the use of fully anonymized residual clinical samples, the requirement for written informed consent was waived in accordance with national regulations and institutional policy.

#### Sample preparation

Slides were scanned into whole slide images (WSIs) at 20× magnification using the Morphogo-U digital slide scanning system (Hangzhou Zhiwei, China). Each WSI encompasses the entire cytology smear, enabling digital image analysis of all cells present.

### Method details

#### Image datasets

The 297 cases were divided into two cohorts: a training set comprising 50 cases and an independent validation set of 247 cases. In total, the cytologists annotated 10,230 non-degenerated urothelial cells from the training cases, comprising 6,648 normal cells, 3,048 atypical cells, and 534 suspicious cells for HGUC according to criteria of TPS.^30^ These expert labels served as ground truth for cell classification model training and five-fold cross-validation. The validation cases included 102 NHGUC cases, 78 AUC cases, 98 SHGUC and HGUC cases and 19 NHGUC-LGUC cases, and they were used exclusively for evaluating the scoring system at WSI level.

#### Cell annotation and segmentation

A semi-automated pipeline was implemented, consisting of candidate cell detection, expert annotation and nuclear-cytoplasmic segmentation. Initially, each WSI was processed by a pre-trained detection model to automatically identify candidate urothelial cells based on morphological characteristics.^12^ Bounding boxes were generated to localize presumptive cell regions.

All detected candidates were subsequently reviewed by two experienced cytologists (SY and CS). Each confirmed urothelial cell was assigned a label—normal, atypical or suspicious for HGUC. Non-urothelial elements and degenerated urothelial cells were excluded during this annotation phase.

Following expert confirmation and labelling, nuclear-cytoplasmic segmentation was performed on each verified cell image using a trained segmentation model. This model was trained on a subset of manually outlined cells and produced a tri-valued masks, assigning each pixel to background, cytoplasm or nucleus. Through this process, accurate compartmentalization of the nucleus and cytoplasm was achieved, enabling reliable morphological and texture feature extraction.

#### Cell feature extraction

Using the segmented cell images, a set of **20 quantitative features** were computed for each cell to characterize its morphology and texture. These features were designed to reflect the diagnostic cytologic criteria defined in TPS.

***Nuclear Area:*** The pixel area of the nucleus, reflecting nuclear size. Abnormal urothelial cells often exhibit enlarged nuclei. TPS specifically defines nuclear size thresholds (e.g. >∼0.3 of an RBC diameter for HGUC).^3^ A larger nuclear area is thus indicative of atypia or malignancy.

***Cell Area:*** The total area of the cell. Along with nuclear size, this gives context to cell size and can identify cells that are unusually large or small.

***Nuclear-to-Cytoplasm Ratio (N/C):*** The ratio of nuclear area to cytoplasmic area. A high N/C ratio is a hallmark of high-grade urothelial carcinoma cells, which tend to have large nuclei and scant cytoplasm. This feature directly encapsulates that cytologic criterion on a continuous scale (range 0 to 1).

***Nuclear Shape Features:*** Nuclear shape irregularity was quantified using three metrics——**nuclear perimeter**, **nuclear roundness** and **nuclear ellipse similarity**. Malignant nuclei often have irregular contours; a lower circularity or disproportionately large perimeter for a given area would suggest membrane irregularities.^2^ We also recorded **nuclear elongation (major axis/minor axis length)** to capture extremely elongated nuclei.

***Cytoplasmic Shape Features:*** Cytoplasmic shape features were assessed through **cytoplasmic roundness** and **cytoplasmic ellipse similarity**, calculated using the same principles as their nuclear counterparts. These metrics reflect the **integrity and symmetry of the whole-cell outline.**

***Nuclear-Cytoplasmic Positional Feature:*** The feature **nuclear-cytoplasmic distance** was computed to measure the displacement of the nucleus from the geometric center of the cytoplasm. To ensure comparability across different cell sizes, the distance was normalized. The nucleus is typically located near the center of the cytoplasm, resulting in **low** nuclear-cytoplasmic **distance** values.

***Chromatin Intensity Features:*** Using the grayscale values of the nuclei converted from the RGB Pap-stained images, **the mean nuclear intensity** and **nuclear intensity standard deviation** were computed to quantify chromatin density and distribution. A higher intensity value corresponds to a lighter nucleus since RGB intensity increases with brightness. Thus, a low mean intensity suggests nuclear hyperchromasia, whereas a high mean intensity may indicate pale-staining nuclei often seen in benign cells. A higher standard deviation in intensity denotes heterogeneous chromatin, consistent with clumping or coarse granularity, while lower variation suggests uniform chromatin. To mitigate inter-slide staining variability, a **normalized nuclear intensity** was also calculated after applying histogram equalization to each cell image. This value reflects nuclear brightness on a standardized scale and helps reduce batch effects caused by variations in staining or scanning.

***Chromatin Texture:*** Beyond first-order intensity stats, we derived second-order texture metrics from the nuclear region using Gray Level Co-occurrence Matrices (GLCM). Specifically, we computed Haralick descriptors including **GLCM energy** and **GLCM contrast**, which quantitatively describe the spatial regularity of pixel intensities. In benign urothelial cells, chromatin is typically fine and evenly distributed, yielding high GLCM energy, indicating homogeneity, and low contrast, reflecting minimal gray-level variation. In contrast, atypical or malignant cells often exhibit coarse, irregular, or clumped chromatin, leading to reduced energy and elevated contrast, as the nuclear texture becomes fragmented and disordered. While GLCM features are mathematically derived, they align closely with the visual assessment by pathologists who evaluate whether chromatin appears “fine” or “coarse” under the microscope.

***Cytoplasmic Intensity Features:*** The **mean intensity** and **intensity variation** of the cytoplasmic region were calculated to describe the overall staining appearance of the cytoplasm. **The mean cytoplasmic intensity** was obtained by averaging grayscale values within the segmented cytoplasm to reflect how strongly the cytoplasm is stained, while **the standard deviation of cytoplasmic intensity** was derived to quantify staining uniformity.

**Edge sharpness:** To assess nuclear structural detail, Canny edge detection was applied to the nuclear region at two threshold settings. **Canny_edge_mean_intensity_10_50** captures weak edge responses using a low threshold. Higher values indicate more fine-grained edges within the nucleus, which may reflect visible chromatin granularity or internal structural irregularities, often seen in atypical cells. **Canny_edge_mean_intensity_50_150** focuses on stronger edges using a high threshold. Higher values suggest clear, coarse nuclear boundaries, such as thickened nuclear membranes or clumped chromatin, features commonly found in malignant cells. Together, these two features help quantify nuclear edge complexity at different scales, offering indirect markers of nuclear atypia and chromatin disruption.

#### Feature distribution analysis and visualization

Each cell was labeled into one of three cytological categories based on cytopathologist review of the WSI: Normal, Atypical, or Suspicious. These categories correspond to increasing levels of nuclear atypia, reflecting the diagnostic gradations in urinary cytology (benign, atypical urothelial cells, and suspicious for malignancy).

Statistical analysis was performed on the extracted features to identify distinguishing characteristics across the three cell categories. For each of the 20 image features, value distributions were visualized using boxplots stratified by cell type (Normal, Atypical, Suspicious). Univariate significance testing was conducted for each feature by computing pairwise two-sample *t*-tests between the classes (Normal vs. Atypical, Normal vs. Suspicious, and Atypical vs. Suspicious). To control for multiple hypothesis testing, Bonferroni correction was applied to the resulting *p*-values. Features with adjusted *p*-values less than 0.05 were considered significantly different between the respective categories.

To further examine global structure in the high-dimensional feature space, *t*-Distributed Stochastic Neighbor Embedding (t-SNE) was applied to reduce the 20-dimensional feature vectors into two dimensions for visualization. Each point in the t-SNE plot represented a single cell, color-coded by its cytological label. This embedding allowed visual inspection of the separability and transition zones between Normal, Atypical, and Suspicious cells, offering insight into the intrinsic continuity of cytological atypia.

#### Model training and evaluation

To classify individual urothelial cells, two ML models were trained using five-fold cross-validation, based on the 20 extracted features. For cross-validation, the annotated cell dataset was randomly partitioned into five equally sized subsets. In each of the five iterations, four subsets were used to train the models, and the remaining subset was used for testing. This process ensured that all annotated cells were evaluated exactly once in the test set and contributed to performance assessment.

An **ordinal logistic regression model (OLR)** was trained to account for the ordinal nature of the categories. All 20 features were included as predictors. The model was fitted on the training set, and its performance was evaluated on the test set. Feature coefficients, odds ratios (ORs), 95% confidence intervals, and *p*-values were computed to assess each feature’s contribution. These effect measures were visualized in a forest plot summarizing the direction and significance of each feature. Model performance was evaluated using a confusion matrix. In parallel, a **random forest classifier (RF)** was trained using the full set of 20 extracted features as input predictors. Feature importance was quantified by the mean decrease in Gini impurity.

Model performance was assessed based on the aggregate predictions across all five folds. The confusion matrices were computed, including sensitivity, specificity, positive predictive value (PPV), negative predictive value (NPV), F1-score, and overall accuracy. The use of cross-validation ensured that performance metrics reflected the model’s generalizability and robustness across the entire dataset.

#### Slide-level scoring and risk analysis

Beyond individual cell classification, we investigated the model’s ability to stratify whole-slide specimens by overall risk of HGUC. To derive slide-level risk indicators, WSIs from the validation set were processed using the full image analysis pipeline. For each WSI, candidate urothelial cells were first detected using the cell detection model, followed by nuclear-cytoplasmic segmentation. The resulting single-cell images were then classified by the trained RF model, which produced probability estimates for each cell belonging to the “Normal,” “Atypical,” or “Suspicious” category.

We then calculated slide-level risk scores by averaging the predicted probabilities of each class across all cells: specifically, the average probability of suspicious urothelial cells on the slide (Suspicious risk), the average probability of atypical urothelial cells on the slide (Atypical risk), and the **combined risk** score defined as the average probability across all detected atypical and suspicious urothelial cells on the slide. Distributions of these three slide-level metrics—suspicious risk, atypical risk, and risk score—were analyzed across four diagnostic categories: HGUC, NHGUC-LGUC, AUC, and NHGUC. Boxplots were generated to visualize the score distributions, and pairwise comparisons were performed using t-tests with Bonferroni correction to assess statistical significance.

### Quantification and statistical analysis

All statistical analyses were performed using Python 3.9 (Python Software Foundation, Wilmington, DE, USA) and GraphPad Prism 10 (GraphPad Software Inc., San Diego, CA, USA). Statistical comparisons of the 20 quantitative cell features among the three cytological categories (Normal, Atypical, and Suspicious) were conducted using two-sample t-tests with Bonferroni correction for multiple comparisons. The exact sample size (n) represents the number of individual annotated urothelial cells (n = 10,230) included in the analysis. Boxplots in Figure 2 display the median and interquartile range (IQR) of each feature, with whiskers extending to 1.5×IQR, representing the distribution and dispersion of the data. Features with adjusted p < 0.05 were considered statistically significant. For dimensionality reduction and visualization of global feature distribution, t-distributed stochastic neighbor embedding (t-SNE) was applied to the 20-dimensional feature vectors (Figure 3). Model training and evaluation were conducted using five-fold cross-validation to ensure robustness. Each cell was tested exactly once, and performance metrics were aggregated across folds. Confusion matrices, shown in Figures 4A and 4C, were used to visualize model predictions, while feature effect estimates and importance measures are presented in Figures 4B and 4D.

Model performance metrics were computed from the cross-validation results, and the metrics were summarized as mean ± standard deviation (SD) across folds. Slide-level risk analyses were performed on 247 validation cases, with group comparisons shown in Figure 5 using boxplots (median and IQR) and two-sample t-tests with Bonferroni correction to determine significance. A p-value less than 0.05 was considered statistically significant for all analyses.
